# The validity of a general factor of *emotional intelligence* in the South African context

**DOI:** 10.4102/ajopa.v5i0.123

**Published:** 2023-03-23

**Authors:** Xander van Lill, Anneke Stols, Pakeezah Rajab, Jani Wiggett

**Affiliations:** 1 Department of Industrial Psychology and People Management, College of Business and Economics, University of Johannesburg, Johannesburg; 2 Department of Product and Research, JVR Africa Group, Johannesburg, South Africa; 3 Department of Psychology, Faculty of Social Sciences, University of East Anglia, Norwich, United Kingdom; 4 Department of Psychology, Faculty of Humanities, University of Pretoria, Pretoria

**Keywords:** Emotional Quotient Inventory 2.0, trait-based emotional intelligence, general factor, individual work performance, employee selection

## Abstract

*Emotional intelligence* (*EI*) plays an important role in the prediction of important work-related outcomes, such as work performance. Southern African scholars frequently deploy total scores of *EI* without considering its hierarchical structure. This study investigated the presence of a general factor, as manifested among the subscales of the EQ-i 2.0, using an archival dataset of 16 581 employees in Southern Africa. Orthogonal first-order, single-factor, higher-order, oblique lower-order and bifactor models were specified to investigate the hierarchical structure of *EI*. The evidence supports the notion that a total score could be calculated for *EI* based on the EQ-i 2.0. A total *EI* score also appears to be predictive of employees’ individual work performance, as measured by their managers. It might, therefore, be practically meaningful for practitioners to calculate or use a total score when making selection decisions about employees based on the EQ-i. 2.0.

**Contribution:**

The findings of the present study offer insights into the theoretical and empirical structure of EI based on statistical techniques that have not been used on the construct in the Southern African context. Concurrent validity evidence further provides additional support that an overall quantitative score, based on the EQ-i. 2.0, has utility in hiring practices, where the aim is to predict future work performance.

## Introduction

Many jobs place high emotional demands on employees; for example, managers or health care workers might be required to – on a daily basis – accurately perceive, understand and regulate their own emotions in the service of fellow employees or patients (Glomb et al., 2004). Employees’ willingness to expend emotional labour or manage their feelings to project a certain public display is becoming increasingly important to performance in many job roles and, ultimately, the social functioning of human enterprise. The relationship between *emotional intelligence* (*EI*) and valued work-related outcomes, such as job performance, is well established. Joseph and Newman (2010) reported a correlation of 0.47 between *EI* and job performance but found a reduced correlation of 0.29 after further refinements in their meta-analytical study (Joseph et al., 2015). A recent meta-analytical study conducted by Sackett et al. (2021) revealed that trait-based *EI* and cognitive ability appeared to be equally relevant predictors of job performance, both with an estimated validity coefficient of 0.30 and 0.31 respectively.

A host of studies on the predictive validity of *EI* has been conducted in South Africa. The most recent study, conducted by Sloan and Geldenhuys (2021), investigated the moderating effect of self-focused *EI* in predicting managers’ *In-role* and *Extra-role performance*, which yielded positive, significant correlations with *EI* of 0.27 and 0.32, respectively. Nel and De Villiers (2004) reported an even higher positive and significant correlation of 0.53 between overall *EI* and job performance in the call centre environment. In contrast to the findings of Sloan and Geldenhuys (2021) and Nel and De Villiers (2004), Hayward et al. (2008) reported a non-significant and negligible effect of *EI* on job performance for managers in a parastatal. However, Hayward et al. (2008) attributed the negligible effect to the limited variance in the performance variable.

While South African research on the predictive validity of *EI* looks promising, there is international debate regarding the legitimacy of using a general factor of *EI*, as performed by Nel and De Villiers (2004) and Hayward et al. (2008), when predicting job performance. A point of concern expressed includes limited investigation of the hierarchical structure of *EI* before a general score is calculated and used to predict work-related outcomes (Dasborough et al., 2021). This problem appears to be endemic to studies conducted in South Africa, with very few investigations conducted on the hierarchical structure of *EI* before inspecting the predictive validity of a general score of *EI*. Van Zyl (2014) conducted the only identifiable study in South Africa that inspected a higher-order model for *EI*, but did not find evidence, in terms of model-data fit based on a confirmatory factor model, to support a general factor of *EI*. Since the study conducted by Van Zyl (2014), specific factor analytical procedures have been recommended by Credé and Harms (2015), which might shed some additional light on the hierarchical structure of *EI* in South Africa. However, before the hierarchical structure of *EI* is addressed, more attention needs to be paid to the theoretical structure of *EI* in this study.

### The theoretical structure of *emotional intelligence*

*Emotional intelligence* is conceptualised differently across various measures, that is, different models are used in its measurement. These models include (1) ability-based *EI* measures, like the Mayer-Salovey-Caruso Emotional Intelligence Test (MSCEIT) (Mayer et al., 2003), (2) self-report (or peer-report) *EI* measures based on the same theoretical model as the MSCEIT (Mayer et al., 2003) and (3) measures of mixed *EI* models, which extend beyond the theoretical model included in the aforementioned two categories (Ashkanasy & Daus, 2005; Dasborough et al., 2021). Such mixed *EI* measures are considered assorted, because they include various items phrased similarly to those used in measuring personality and behavioural preferences (Ashkanasy & Daus, 2005). In contrast to the ability-based *EI* measured by the MSCEIT (Mayer et al., 2003), the self-report *EI* measures from the two latter models are often termed ‘trait-based *EI*’, as it has to do with individuals’ perceptions of their own emotional skills (Joseph et al., 2015; Petrides et al., 2016). All three of these models include scientifically sound *EI*-related constructs that could greatly contribute to what we know about work performance (Dasborough et al., 2021; Joseph et al., 2015).

Within the categories of ability-based *EI* and trait/mixed *EI*, the two theoretical models that have received a lot of attention when exploring the relationships between *EI* and job performance were the models that underpin the MSCEIT (Mayer et al., 2003) and the Bar-On Emotional Quotient Inventory (Joseph et al., 2015). This article focuses on the revised version of the latter model, namely the EQ-i 2.0 model (Wiechorek, 2011).

The EQ-i 2.0 model was established based on 25 years of research on the different aspects that constitute *EI*, including how these interrelate. The EQ-i 2.0 model has a multidimensional structure that provides an indication of a person’s total *EI*, which is contextualised by the different facets believed to underlie *EI*. The total *EI* score reflects a ‘snapshot’ of a person’s overall *EI* and can be defined as (Wiechorek, 2011):

[*A*] set of emotional and social skills that influence the way we perceive and express ourselves, develop and maintain social relationships, cope with challenges, and use emotional information in an effective and meaningful way. (p. 49)

**FIGURE 1:**
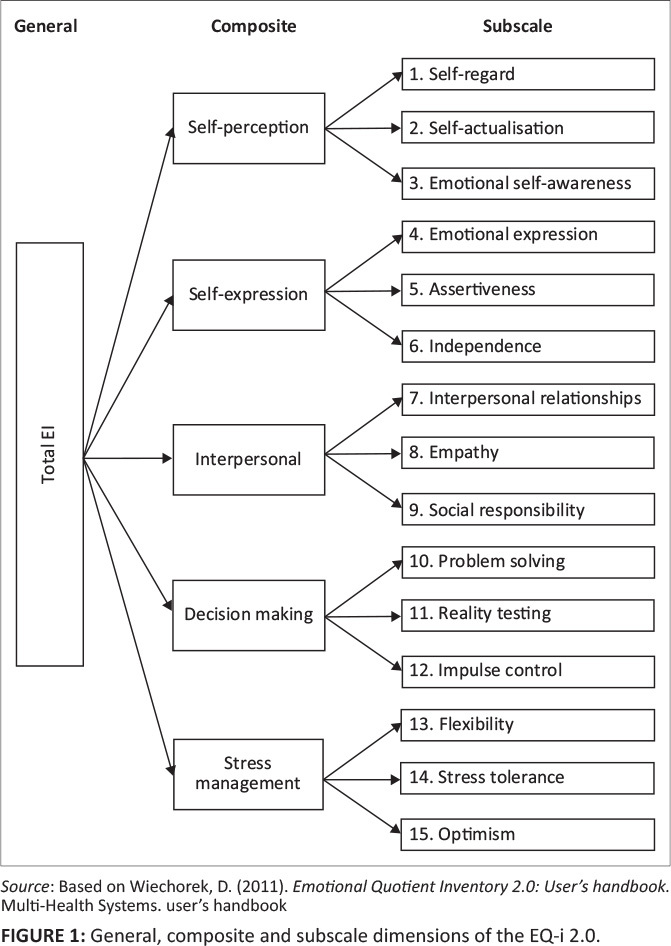
General, composite and subscale dimensions of the EQ-i 2.0.

This broad definition also speaks to the different facets included in the EQ-i 2.0, which encompass 15 constructs that can be collapsed into five comparable categories or composites. A visual depiction of the theoretical structure of the EQ-i 2.0, including the proposed general factor, is presented in Figure 1.

The meaning of the composites and subscales’ measures, as demonstrated in Figure 1, is described in the Wiechorek (2011) user’s handbook. The first three subscales, noted below, collectively quantify an individual’s Self-perception, which describes how people see themselves:

1. **Self-regard:** Having self-respect and confidently accepting one’s gifts and flaws.
2. **Self-actualisation:** Consistently working to better oneself or to reach goals of importance.
3. **Emotional self-awareness:** Understanding one’s emotions and how they affect oneself.

The second, the Self-expression composite scale, considers how people express their inner perception of themselves, which is jointly portrayed through the subscales noted below:

4. **Emotional expression:** To share one’s emotions in a constructive way.
5. **Assertiveness:** To respectfully communicate one’s feelings and views.
6. **Independence:** Relying on oneself and not depending on others emotionally.

The Interpersonal composite scale considers the nature of the relationships that people build with others. The subscales that collectively inform this composite are:

7. **Interpersonal relationships:** To form and uphold trusting relationships that are agreeable to all parties.
8. **Empathy:** Recognising and understanding others’ feelings and showing concern for them.
9. **Social responsibility:** Being socially conscious and helpful towards others in the community.

The Decision making composite scale considers how people use emotional information to effectively make decisions, which is jointly established through:

10. **Problem-solving:** Understanding how one’s emotions impact decisions and solving problems despite these emotions.
11. **Reality testing:** To be aware of the reality of a situation and display one’s objectivity.
12. **Impulse control:** Being able to withstand an urge to act or make rash decisions.

The Stress management composite scale measures peoples’ ability to deal with stressors, utilising multiple coping strategies and showing resilience despite setbacks and incorporates:

13. **Stress tolerance:** To cope with and manage stressful situations to achieve a positive outcome.
14. **Flexibility:** To flexibly adapt one’s actions, feelings and thoughts to change.
15. **Optimism:** To remain positive and resilient despite facing obstacles.

The scales, measured at every level of the EQ-i 2.0, were designed with a specific function in mind. The subscales serve to provide a more foundational understanding of employees’ relative strengths and weaknesses, which is valuable for development purposes. Compared to a general factor, subscales are qualitatively more meaningful during psychometric feedback. Psychometric feedback on subscales provides the opportunity to suggest actionable steps that employees could take to increase their overall *EI* at work. An *EI* total, or even a composite *EI* score, might be perceived as too ambiguous and less meaningful from a development perspective. By contrast, the composite and overall dimensions of the structure might provide more encompassing, and therefore also more consistent, dimensions that can be utilised for selection purposes (Wiechorek, 2011).

A study conducted by Van Zyl (2014) supported the existence of the composites, as set out in Figure 1. Van Zyl (2014) was, however, unable to find support for a general factor of *EI* among the subscales of the EQ-i 2.0. However, Van Zyl (2014) did not inspect the recently suggested sequence for the inspection of hierarchical structure (Credé & Harms, 2015), which was explored in the current study. The present researchers were particularly interested in the general factor of *EI* as based on subscales of the EQ-i 2.0 and specified a bifactor model.

### The theoretical structure of individual work performance

Van Lill and Taylor’s (2022) framework underlying the Individual Work Performance Review (IWPR) was utilised to conceptualise and measure performance in the present study. Five broad performance dimensions are differentiated by Van Lill and Taylor (2022), including *in-role, extra-role, adaptive, leadership* and *counterproductive performance*. According to Van Lill and Taylor (2022, pp. 3–5):

1. *In-role performance* refers to: ‘Actions that are official or known requirements for employees (Carpini et al., 2017; Motowidlo & Van Scotter, 1994). These behaviours could be viewed as the technical core (Borman & Motowidlo, 1997) that employees must demonstrate to be perceived as proficient and able to contribute to the achievement of organisational goals’ (Carpini et al., 2017).
2. *Extra-role performance* refers to: ‘future- or change-orientated acts (Carpini et al., 2017), aimed at benefitting co-workers and the team (Organ, 1997), that are discretionary or not part of the employee’s existing work responsibilities’ (Borman & Motowidlo, 1997).
3. *Adaptive performance* relates to: ‘employees’ demonstration of the ability to cope with and effectively respond to crises or uncertainty’ (Carpini et al., 2017; Pulakos et al., 2000).
4. *Leadership performance* refers to: ‘the effectiveness with which an employee can influence co-workers to achieve collective goals’ (Campbell & Wiernik, 2015; Hogan & Sherman, 2020; Yukl, 2012).
5. *Counterproductive performance* reflects on the: ‘intentional or unintentional acts (Spector & Fox, 2005) by an employee that negatively affect the effectiveness with which an organisation achieves its goals and cause harm to its stakeholders’ (Campbell & Wiernik, 2015; Marcus et al., 2016).

**FIGURE 2:**
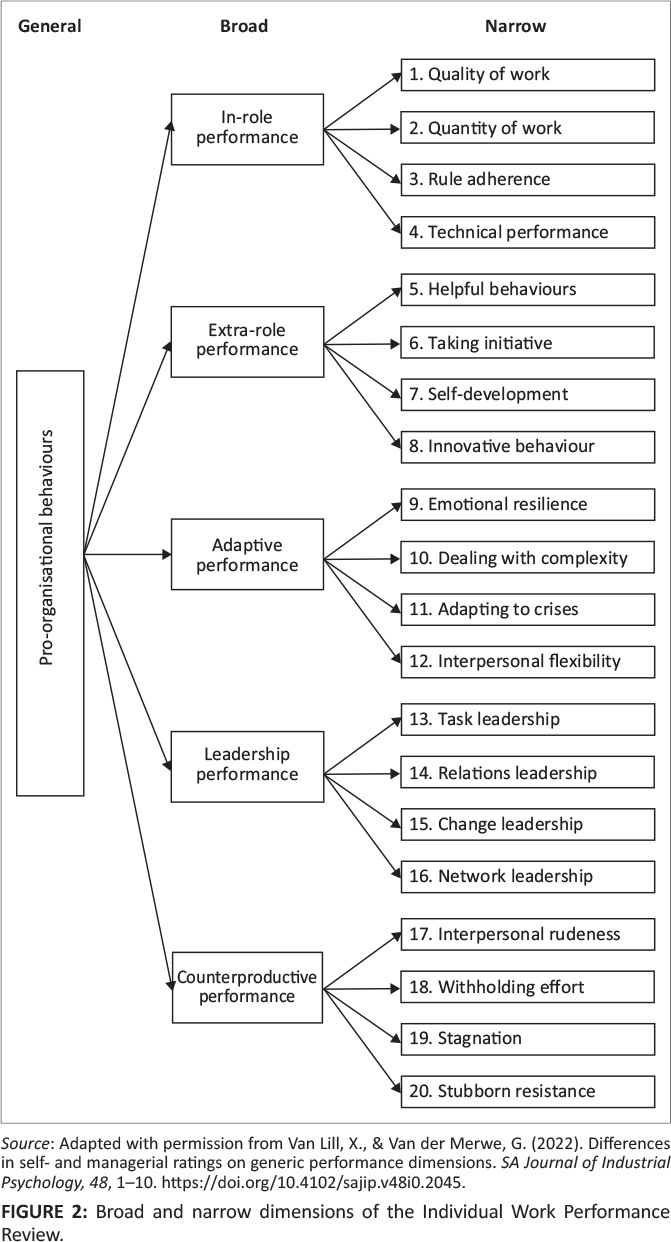
Broad and narrow dimensions of the Individual Work Performance Review.

Each of the five broad performance dimensions is represented by four narrow performance dimensions, as shown in Figure 2. Evidence in support of the five factors and definitions of the narrow dimensions can be obtained from Van Lill and Taylor (2022). As portrayed in Figure 2, it is theorised that a general factor stands at the apex of all the performance dimensions identified in the IWPR. Van Lill and Van der Vaart (2022) found that a general factor explained a similar amount of variance in South Africa as that reported by Viswesvaran et al.’s (2005) meta-analytical study. The present study focused on the predictive validity of total *EI* for general and broad dimensions of individual work performance.

### Predictive validity of a general factor of *emotional intelligence*

The evidence suggests that the criterion validity of *EI* is replicable in the South African context (Nel & De Villiers, 2004; Sloan & Geldenhuys, 2021). However, it is less clear what specific work-related behaviours *EI* predicts when compared to more established international scientific literature. Performance is a multidimensional construct, and Van Lill and Taylor (2022) suggest a five-factor model for individual behaviours at work, namely in-role, extra-role, adaptive, leadership and counterproductive performance. The only local evidence that differentiated between performance dimensions was the study conducted by Sloan and Geldenhuys (2021), which focused on both in-role and extra-role performance as work-related outcomes.

Meta-analytical evidence to date suggests that *EI* is predictive of in-role performance, also referred to as ‘task performance’ (Joseph et al., 2015; Sackett et al., 2021). Emotional self-regulation is more frequently recognised as a core part of functioning in social enterprises and, therefore, the ability to succeed at essential tasks (Joseph et al., 2015).

There is also evidence in support of the relationship between *EI* and extra-role performance. Employees with higher *EI* might be more empathetic and prosocial, which could, in turn, lead to greater displays of extra-role performance or actions aimed at doing more than what is required by their job descriptions (Miao et al., 2017).

*Emotional intelligence* might also assist employees in better coping with negative emotions that arise from interpersonal strain or frustrating tasks. Consequently, they are less inclined to engage in deviant intrapersonal or interpersonal behaviours that undermine collective goals (Miao et al., 2017).

It further appears that *EI* could assist individuals in coping with the strain associated with the complexities of change at work. In this respect, *EI* could help individuals to downregulate negative emotions in response to uncertainty and help them increase positive feelings, in order to stay focussed on solutions in response to change. *Emotional intelligence* is, therefore, also argued to be related to adaptive performance (Yang et al., 2022).

Finally, *EI* might translate into greater self-confidence, self-awareness and empathy, which are essential components of interpersonal influence and, therefore, leadership performance (Harms & Credé, 2010). An overview of the evidence presented suggests that *EI* is likely to be related to all five performance dimensions, namely in-role, extra-role, adaptive, leadership and counterproductive performance.

The EQ-i 2.0 has been used across multiple industries in the United States of America, with accumulating evidence of the instrument’s utility in differentiating between high- and low-performing individuals (Stein & Book, 2011). However, limited research has been conducted on the predictive validity of the general factor of the EQ-i 2.0 in South Africa, which was one of the areas of focus of the current study.

### Research objective and hypotheses

The objective of this study was to determine the structural and criterion validity of a general dimension of *EI* in the EQ-i 2.0 assessment. Based on the current evidence reported in the present study, the following hypotheses were formulated:

#### Study 1

**H_1_:** The general factor of *EI* explains covariance between the items, independent of the covariance that the 15 facets explain in the same set of items.

#### Study 2

**H_2_**: General *EI* has a significant positive effect on overall job performance.

**H_2A_**: General *EI* has a significant positive effect on in-role performance.

**H_2B_**: General *EI* has a significant positive effect on extra-role performance.

**H_2C_**: General *EI* has a significant positive effect on adaptive performance.

**H_2D_**: General *EI* has a significant positive effect on leadership performance.

**H_2E_**: General *EI* has a significant negative effect on counterproductive performance.

## Method

### Participants

For Study 1, a sample of 16 581 working adults living in Southern Africa was obtained via an online platform. The mean age of the respondents was 37.94 years (standard deviation [SD] = 8.86). Most respondents self-identified as male (*n* = 9427; 57%), followed by females (*n* = 7154; 43%). The sample further included individuals who indicated their ethnicity as follows: black (*n* = 6755; 41%), white (*n* = 4915; 30%), coloured (mixed ancestry; *n* = 1675; 10%) and Indian or Asian (*n* = 1838; 11%). The researchers computed the power for the test model (degrees of freedom [*df*]= 6667), based on the computer software developed by Preacher and Coffman (2006). The models returned a value of unity, which suggested that an incorrect model would be correctly rejected (α = 0.05; null root mean square error of approximation [RMSEA] = 0.05; alternative RMSEA = 0.08).

For Study 2, a total of 108 performance ratings of South African employees, who were also administered the EQ-i 2.0, were completed by managers in two participating organisations, selected via a census or stratified sampling strategy. The sample represented the finance and professional services sectors. The mean age of employees was 38.88 years (SD = 7.78 years). Most of the employees self-identified as white (*n* = 65; 60%), followed by black African (*n* = 18; 17%), Indian (*n* = 16; 15%), coloured (individuals of mixed ancestry; *n* = 8; 7%) and Asian (1; 1%). The sample comprised more women (*n* = 65; 60%) than men (*n* = 43; 40%). Most of the employees were registered professionals (*n* = 53; 49%), followed by low-level managers (25; 23%), mid-level managers (*n* = 16; 15%), skilled employees (*n* = 12; 11%) and top-level managers (2; 2%). The present researchers inspected the statistical power required for linear bivariate regression by using G*Power (Faul et al., 2007). The calculation suggested that 64 participants should be sufficient (α = 0.05; Power = 0.80) to detect a slope of 0.30, per prior meta-analytical validity estimates reported (Sackett et al., 2021). The present sample was roughly double the recommended size based on this calculation.

### Instruments

The EQ-i 2.0 assessment consists of 133 items; that is an average of eight items in each of the 15 subscales (i.e. three subscales per composite). Eight items contribute to a *Well-being* indicator, also referred to as the ‘happiness scale’, while seven of these items are also used as a validity measurement. The EQ-i 2.0 model comprises a 1-5-15 structure, where the 15 subscales underlie the five composites that all contribute to one total *EI* ‘snapshot’ (see Figure 1). A five-point Likert-type frequency scale provides the response options for each item. The response options range from 1 = Never/Rarely to 5 = Almost Always/Always, with a qualitative interpretation guide connected to the meaning of each option (Wiechorek, 2011). The internal consistency reliabilities for the South African sample on most of the EQ-i 2.0 subscales were satisfactory (α and ω ≥ 0.71). Only one subscale had an unsatisfactory internal consistency reliability coefficient of 0.66, namely independence. However, this reliability coefficient was still considered marginally acceptable (see Table 1).

**TABLE 1:** Descriptive statistics for the EQ-i 2.0 subscale factors.

EQ-i 2.0 subscale factors	SR	SA	ES	EE	AS	IN	IR	EM	SO	PS	RT	IC	FL	ST	OP
Self-regard (SR)	-	0.79	0.58	0.53	0.71	0.64	0.61	0.33	0.58	0.70	0.70	0.46	0.56	0.67	0.78
Self-actualisation (SA)	0.78*	-	0.70	0.49	0.77	0.60	0.66	0.49	0.76	0.70	0.86	0.43	0.55	0.75	0.80
Emotional self-awareness (ES)	0.57*	0.69*	-	0.60	0.66	0.39	0.58	0.75	0.64	0.50	0.94	0.43	0.45	0.56	0.60
Emotional expression (EE)	0.52*	0.47*	0.59*	-	0.55	0.34	0.65	0.52	0.52	0.50	0.51	0.35	0.59	0.39	0.51
Assertiveness (AS)	0.70*	0.76*	0.64*	0.53*	-	0.60	0.57	0.40	0.61	0.65	0.79	0.34	0.43	0.67	0.62
Independence (IN)	0.62*	0.59*	0.37*	0.33*	0.59*	-	0.31	0.17	0.35	0.85	0.55	0.56	0.64	0.67	0.49
Interpersonal relationships (IR)	0.60*	0.64*	0.57*	0.64*	0.56*	0.29*	-	0.65	0.70	0.43	0.62	0.27	0.52	0.53	0.65
Empathy (EM)	0.31*	0.47*	0.74*	0.51*	0.38*	0.15*	0.64*	-	0.67	0.28	0.64	0.34	0.37	0.39	0.52
Social responsibility (SR)	0.57*	0.75*	0.62*	0.51*	0.60*	0.33*	0.69*	0.65*	-	0.48	0.68	0.35	0.48	0.55	0.67
Problem solving (PS)	0.69*	0.68*	0.48*	0.48*	0.64*	0.84*	0.41*	0.26*	0.47*	-	0.68	0.72	0.75	0.84	0.61
Reality testing (RT)	0.69*	0.85*	0.93*	0.50*	0.78*	0.53*	0.61*	0.63*	0.67*	0.66*	-	0.52	0.51	0.77	0.70
Impulse control (IC)	0.45*	0.42*	0.41*	0.33*	0.32*	0.54*	0.25*	0.32*	0.34*	0.71*	0.50*	-	0.56	0.55	0.42
Flexibility (FL)	0.55*	0.54*	0.44*	0.58*	0.42*	0.63*	0.50*	0.36*	0.46*	0.73*	0.50*	0.55*	-	0.66	0.58
Stress tolerance (ST)	0.66*	0.74*	0.55*	0.38*	0.66*	0.66*	0.51*	0.38*	0.54*	0.83*	0.76*	0.54*	0.65*	-	0.68
Optimism (OP)	0.77*	0.79*	0.59*	0.50*	0.60*	0.47*	0.64*	0.51*	0.65*	0.59*	0.69*	0.40*	0.57*	0.67*	-
Mean	4.47	4.60	4.26	3.87	4.14	4.31	4.29	4.14	4.28	4.46	4.28	4.26	3.85	4.29	4.45
*SD*	0.49	0.38	0.52	0.69	0.49	0.46	0.52	0.55	0.53	0.49	0.44	0.53	0.56	0.53	0.47
Alpha	0.90	0.90	0.85	0.88	0.79	0.77	0.88	0.90	0.81	0.88	0.84	0.81	0.79	0.89	0.88
Omega	0.81	0.83	0.80	0.86	0.71	0.66	0.83	0.86	0.77	0.81	0.77	0.74	0.74	0.85	0.83

Note: Upper limit is reported above the diagonal, and inter-factor correlations are below the diagonal.

SD, standard deviation.

* , *p* < 0.01

The IWPR (Van Lill & Taylor, 2022) consists of 80 items (4 items for each of the 20 narrow performance dimensions) that cover five factors, namely *In-role performance, Extra-role performance, Adaptive performance, Leadership performance* and *Counterproductive performance* (Van Lill & Taylor, 2022). Per the guidelines of Aguinis (2019), each item was measured using a five-point behavioural frequency scale. Word anchors defined the extreme points of each scale, namely, 1 = Never demonstrated and 5 = Always demonstrated. Qualitative interpretation of numeric values between the extreme points is provided, to better approximate an interval rating scale, namely 2 = Rather infrequently demonstrated, 3 = Demonstrated some of the time and 4 = Quite often demonstrated. Van Lill and Taylor (2022) demonstrated the internal consistency reliability of all the narrow dimensions of the IWPR (α and ω ≥ 0.83).

### Procedure

The data on the EQ-i 2.0 (*n* = 16 581) were collected as part of several archival projects, for different client projects on the JVR Online platform. Data were collected via online assessments for either selection or development purposes. A concurrent set of data was separately collected by asking managers of the 108 employees, who simultaneously completed the EQ-i 2.0, to rate their employees’ performance. A study conducted by Van Lill and Van der Merwe (2022) revealed that employees greatly inflate self-ratings on the IWPR (Van Lill & Taylor, 2022) when compared to managerial ratings, because of leniency bias. Managers might therefore provide a more conservative and accurate estimate of work performance (Van Lill & Van der Merwe, 2022). Managerial ratings also come with the added benefit of reduced method bias, because of another rating source used in addition to self-ratings on the EQ-i 2.0 (Podsakoff et al., 2012).

### Data analysis

#### Study 1: Confirmatory factor analysis

**FIGURE 3:**
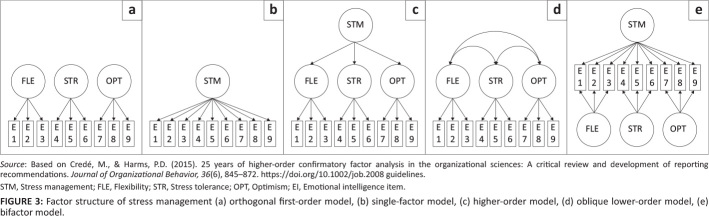
Factor structure of stress management (a) orthogonal first-order model, (b) single-factor model, (c) higher-order model, (d) oblique lower-order model, (e) bifactor model.

Confirmatory factor analysis (CFA) was performed using Version 0.6–12 of the lavaan package (Rosseel, 2012; Rosseel et al., 2022) in R (R Core Team, 2016) to first inspect the inter-factor correlations between all the narrow *EI* factors, whereafter the hierarchical factor structure of the broad performance factors was investigated. A prior study conducted a higher-order factor analysis to inspect the general factor of *EI* in the EQ-i 2.0 (Van Zyl, 2014). Recent best practice guidelines recommend testing a sequence of five models before the presence of hierarchical structure of a psychometric measure is confirmed or refuted, namely (1) orthogonal first-order, (2) single-factor, (3) higher-order, (4) oblique lower-order and (5) bifactor models (Credé & Harms, 2015). A visual example of the different factor models is portrayed for composite Stress Management in Figure 3. Single-factor models (all items load on one factor) and orthogonal first-order models (factor models with uncorrelated lower-order factors) represent parsimonious models, and, if these models display greater fit, it might discredit the existence of hierarchical structure in the data. By contrast, better fit for lower-order (factor models with correlated factors), higher-order (items load on lower-order factors, which, in turn, load onto second-order factors) and bifactor models (items are specified simultaneously on uncorrelated first- and second-order factors) provides more support for hierarchical structure (Credé & Harms, 2015).

First-order factors, as specified in higher-order models, mediate the relationship between manifest variables and second-order factors and, therefore, do not explain unique variance in the manifest variables over and above the *EI* subscales (Beaujean, 2014; McAbee et al., 2014). Bifactor models differ in this respect by accounting for the unique variance that a general factor explains in the manifest variables, beyond the variance explained by the uncorrelated lower-order *EI* subscales (Beaujean, 2014; McAbee et al., 2014). Bifactor models were, therefore, used to test the existence of a general factor in *EI*, as manifested among the subscales of the EQ-i 2.0.

Diagonally weighted least squares (DWLS) estimation, with robust standard errors, was performed to inspect the hierarchical factor structure of *EI* (DiStefano & Morgan, 2014; Li, 2016). This method provides accurate estimates when larger samples are used (*n* > 500) when the data are multivariate non-normal, and is less sensitive when the data are based on an ordinal rating scale with five or more categories (DiStefano & Morgan, 2014; Li, 2016). The multivariate skewness (1234726.00; *p* < 0.001) and kurtosis (1200.95, *p* < 0.001) for the entire set of 118 items suggested that the data were non-normally distributed. Model-data fit was considered acceptable if the RMSEA and standardised root mean squared residual (SRMR) were ≤ 0.08 (Brown, 2015; Browne & Cudeck, 1992), and the comparative fit index (CFI) and Tucker–Lewis index (TLI) were > 0.95 (Brown, 2015; Hu & Bentler, 1999). Even when CFIs display a marginally good fit to the data (CFI and TLI in the range of 0.90 to 0.95), models might still be considered to display an acceptable fit if other indices (SRMR and RMSEA) are also within the acceptable range (Brown, 2015).

#### Study 2: Regression analysis

The researchers conducted separate linear regressions by means of the lm function in R (R Core Team, 2016). For the different models, a summed raw total *EI* score was regressed on a general dimension, as well as separate broad dimensions, of *Individual work performance*.

### Ethical considerations

The current study was low in risk, but precautions were taken to ensure that participation was voluntary and anonymous, that no harm was caused, that the questions were filled in truthfully and that informed consent was given to use the results for research purposes.

Ethical clearance to conduct this study was obtained from the University of Johannesburg Department of Industrial Psychology and People Management Research Ethics Committee (reference number: IPPM-2022-598).

## Results

### Study 1: Confirmatory factor analysis

The mean item score and SD for each subscale of the EQ-i 2.0, along with the alpha and omega reliability estimates and standardised inter-factor correlations, are reported in Table 1. The inter-factor correlations were obtained by conducting an oblique lower-order confirmatory factor model. The fit statistics for the oblique lower-order confirmatory factor model of the entire EQ-i 2.0 (χ^2^ [*df*] = 254827.62 [6680]; CFI = 0.97; TLI = 0.97; SRMR = 0.05; RMSEA = 0.05 [0.05; 0.05]) were satisfactory (Brown, 2015). The oblique lower-order model, which fit statistics are also reported in Table 2, allows group factors to covary and enables an inspection of the inter-factor correlations between the dimensions for descriptive purposes.

**TABLE 2:** Fit statistics of different EQ-i 2.0 factor models.

Model	χ^2^	Δ χ^2^	*df*	CFI	TLI	SRMR	RMSEA
Test	393095.62	-	6667	0.95	0.95	0.07	0.06 (0.06; 0.06)
4	444753.71	5636964.10*	6770	0.94	0.94	0.07	0.06 (0.06; 0.06)
3	254827.62	5585306.01	6680	0.97	0.97	0.05	0.05 (0.05; 0.05)
2	756321.23	5775232.10*	6785	0.91	0.90	0.08	0.08 (0.08; 0.08)
1	6030059.72	5273738.49*	6785	0.23	0.22	0.26	0.23 (0.23; 0.23)

Note: Model 1, orthogonal first-order model; Model 2, single-factor model; Model 3, oblique lower-order model; Model 4, higher-order model and Test (model compared with) = bifactor model. Values reported in brackets represent the lower and upper limit of RMSEA confidence intervals.

*df*, degrees of freedom; RMSEA, root mean square error of approximation; SRMR, standardised root mean squared residual CFI, comparative fit index; TLI, Tucker–Lewis index.

* , *p* < 0.01.

The size of the relationships reported in Table 1 was mostly in the medium-to-large range, alluding to the possibility of a general factor. However, 95% of the standardised upper limit inter-factor correlations (UL) were below the cut-off recommended by Rönkkö and Cho (2020), namely UL < 0.80, suggesting a fair degree of discriminant validity at the subscale level. In a select few cases (5% of UL), insufficient evidence for discriminant validity existed. However, the lower levels of discriminant validity could be attributable to established theoretical relationships that have been reported between the scales in the past (Rönkkö & Cho, 2020; Wiechorek, 2011).

Table 1 also contains the inter-item consistency reliabilities. All the subscales obtained coefficient alpha ordinal and McDonald’s omega values (α and ω ≥ 0.71) above the recommended threshold (Cortina, 1993; Cortina et al., 2020). Only the independence subscale had an ω-value of 0.66, which was still accepted, as the α ordinal value was 0.77. These results suggest that the subscales reliably measure the respective scale constructs.

The fit of different factor models proposed by Credé and Harms (2015) was subsequently investigated to determine whether a general factor or alternative configurations explained the covariances between the subscale dimensions of the EQ-i 2.0. The different models are reported in Table 2.

The results reported in Table 2 indicated that the more parsimonious models, namely the orthogonal first-order and single-factor models, displayed a weaker fit to the data (Credé & Harms, 2015). By contrast, the more complex models (oblique lower-order, higher-order and bifactor models) displayed superior fit, supporting the existence of hierarchical structure in the data. The present study focussed on the manifestation of a general factor of *EI* and, therefore, necessitated a further inspection of the satisfactory fitting bifactor model, instead of the slightly better fitting oblique lower-order factor model.

It is recommended that bifactor statistical indices be calculated to determine the practical meaningfulness of general versus group factors in a bifactor analysis (Rodriguez et al., 2016a, 2016b), such as the explained common variance (ECV), coefficient omega hierarchical (ω_h_), construct replicability (H), factor determinacy (FD), percentage uncontaminated correlations (PUC) and relative percentage bias (ARPB). Group factors are considered plausible when ω_h_, H and FD^2^ are > 0.50, 0.70 and 0.70, respectively (Dueber, 2017; Reise et al., 2013). Explained common variance for the general factor > 0.70 and PUC > 0.80 are indicative of unidimensionality (Reise et al., 2013). Relative percentage bias below 10% – 15% indicates little difference in the factor loadings between a single-factor model and the general factor in a bifactor model (Rodriguez et al., 2016a). Bifactor statistical indices were calculated using Version 0.2.0 of the Bifactor Indices Calculator package (Dueber, 2020) in R (R Core Team, 2016). The bifactor statistical indices are reported in Table 3.

**TABLE 3:** Bifactor statistical indices for EQ-i 2.0 subscale factors.

Factors and facets	ECV	Omega	OmegaH	H	FD
**General EI**	**0.58**	**0.97**	**0.91**	**0.98**	**0.98**
Self-regard	0.03	0.82	0.27	0.66	0.86
Self-actualisation	0.02	0.85	0.16	0.54	0.79
Emotional self-awareness	0.02	0.78	0.26	0.59	0.81
Emotional expression	0.04	0.81	0.44	0.77	0.90
Assertiveness	0.02	0.74	0.30	0.61	0.82
Independence	0.03	0.70	0.43	0.67	0.83
Interpersonal relationships	0.03	0.81	0.30	0.74	0.89
Empathy	0.06	0.86	0.54	0.83	0.93
Social responsibility	0.02	0.74	0.27	0.63	0.85
Problem solving	0.02	0.81	0.28	0.63	0.83
Reality testing	0.01	0.77	0.14	0.43	0.70
Impulse control	0.04	0.73	0.51	0.77	0.89
Flexibility	0.03	0.72	0.36	0.64	0.83
Stress tolerance	0.03	0.86	0.29	0.66	0.88
Optimism	0.02	0.87	0.25	0.67	0.91

Note: Relative parameter bias (ARPB) for General EI = 0.05; PUC for General EI = 0.94. Values in bold reflect estimates that are based on the general factor of *EI*.

EI, Emotional intelligence; ECV, explained common variance; FD, factor determinacy.

The *General EI* factor accounted for over half of the common variance. The ECV > 0.50, including the high PUC > 0.80, suggests the presence of a strong general factor (Reise et al., 2013). These results were further supported by the ARPB value of 5%, which was well below the 10% – 15% mark, indicating no serious concern of measurement bias if the model was treated as unidimensional (Rodriguez et al., 2016a).

The subscale values all had a large difference between the ω and ω_h_ values, with ω_h_ ranging from 0.14 to 0.54. This normally suggests that the subscales mostly do not add additional unique variance, and that the factor model is unidimensional. However, Morin (2023) cautions against the use of ω and ωhs, as both tend to underestimate the reliability of group factors. The general trend of the FD and H coefficients, as well as the evidence of the discriminant validity of the group factors presented in Table 1, suggests that the subscales in the EQ-i 2.0 add additional interpretive value for development purposes (Dueber, 2017; Reise et al., 2013). The subscales also still explain the remaining 42% of the variance in the items. Consequently, at a cursory view of the hierarchical structure and without discounting the value of the subscales, it can be argued that a general *EI* factor exists, which supports Hypothesis 1.

### Study 2: Regression analysis

Linear regressions were conducted to determine the relationship between overall *EI* and individual work performance. The regression coefficients of the different relationships are reported in Table 4.

**TABLE 4:** Total *emotional intelligence* regressed on general and broad dimensions of individual work performance.

EI regressed on:	*R*	*R* ^2^	Adj. *R^2^*	Δ *F*	df1	df2	Δ Sig. *F*
General	0.39	0.15	0.14	18.44	1	106	< 0.001
In-role	0.33	0.11	0.10	13.20	1	106	< 0.001
Extra-role	0.25	0.06	0.06	07.29	1	106	< 0.001
Adaptive	0.37	0.14	0.13	17.10	1	106	< 0.001
Leadership	0.42	0.18	0.17	24.40	1	106	< 0.001
Counterproductive	−0.36	0.13	0.12	15.98	1	106	< 0.001

*R*, correlation coefficient; *R*^2^, regression coefficient; Adj. *R*^2^, regression coefficient adjusted for number of predictors; Δ*F*, comparison between current regression model to model with no independent variable; *df* 1, numerator degrees of freedom; *df* 2, denominator degrees of freedom; Δ Sig. *F*, statistical significance of change in F-statistic; EI, Emotional intelligence; *df*, degrees of freedom.

In comparison to meta-analytical evidence, the validity estimate (*R* = 0.39) for *General performance* appears to be slightly higher in the present study compared to other meta-analytical estimates, namely ρ = 0.29 (Joseph et al., 2015) and ρ = 0.30 (Sackett et al., 2021). However, the estimate was still in the same direction and, similar to prior findings, moderate in size. Hypothesis 2 was, therefore, confirmed. The correlations for the broad dimensions of performance also appeared mostly moderate in size (*R* = 0.25 to 0.42; M = 0.35) and in the theorised directions. Therefore, Hypotheses 2A to 2E could also be confirmed. The regression coefficient for *Leadership performance* appeared to be pronounced, which could be attributed to the high emotional labour often associated with interpersonal influence (Glomb et al., 2004).

## Discussion

Study 1 supported the existence of a strong general factor of *EI*, which explained 58% of the common variance. Practically, the findings suggest that a total score of *EI*, based on the EQ-i 2.0, could be calculated. A total score might enable scientists to include an overall *EI* score in regression analyses. A total score could also aid practitioners in including an overall *EI* score in selection decisions, especially when other psychometric results must be considered simultaneously. However, a nuanced interpretation based on the subscales still has merit, as it further ‘colours’ a person’s strengths and weaknesses, especially for use in development feedback and work-based counselling. An overall *EI* score might come across as more ambiguous and less actionable from a development perspective, compared to a more nuanced interpretation (Wiechorek, 2011).

In terms of Study 2, total *EI* appeared to have a pattern of relations with performance that replicates international results on the predictive validity of *EI*. Employees who display a fair degree of *EI* might be valued by supervisors as stable, well-functioning individuals and therefore be perceived as high performers. More specifically, *EI* could be a valuable psychological resource in uncertain conditions, such as the strain placed on employees during the coronavirus disease 2019 pandemic (Moroń & Biolik-Moroń, 2021). In such conditions, based on the findings of the present study, *EI* might assist employees in adapting to change (*Adaptive performance*) and initiate the necessary interpersonal influence (*Leadership performance*) to ensure that organisational goals are achieved, whether within or away from the office setting. The predictive validity of total *EI* for one of the most valued work-related outcomes in psychology, namely individual work performance (Campbell & Wiernik, 2015), gives credence to the utility of such a score in employee selection processes.

### Limitations and recommendations for future research

Mean group gender differences have been reported for *EI* in the past, with scholars suggesting that men might be adversely affected in selection procedures if a total score of *EI* is used in isolation to make a hiring decision (Joseph & Newman, 2010). Multi-group CFAs suggest that composite scales in the EQ-i 2.0 are invariant across gender groups. An inspection of mean group differences further revealed only small effect size differences (Stols & Van Lill, 2022). Further research could inspect the invariance and mean group gender differences of a general dimension of *EI* based on the EQ-i 2.0 in the Southern African context.

The predictive validity of total *EI* was mainly based on professional and managerial staff, many of whom were employed in the financial and health sector, for whom high *EI* might be a more pronounced occupational requirement. Most other Southern African studies appeared to have sampled managerial employees, and future studies could inspect whether these relationships are replicable for other job families in South Africa, such as skilled/semiskilled, clerical, military and law enforcement jobs.

## Conclusion

Prior studies in South Africa frequently used total scores on *EI* to predict work-related outcomes without considering the hierarchical structure of the measure (Hayward et al., 2008; Nel & De Villiers, 2004; Sloan & Geldenhuys, 2021). This study inspected and presented evidence in favor of the calculation of a general factor of *EI* based on the EQ-i 2.0. The evidence further suggests that the total *EI* score, in accordance with the findings of Nel and De Villiers (2014) and Sloan and Geldenhuys (2021), yields meaningful validity estimates for work-related outcomes, such as work performance. Consequently, using the total *EI* score in a report might be meaningful when, for example, selection decisions need to be made about employees.
